# Supplementary material for: Spatial Variation in Stream-Water Bacterial Communities Across Sites Differing in Wild Chinese Giant Salamander Detection Frequency
Source: Biology (Basel). 2026 Sep 18;15(18):1647. doi: 10.3390/biology15181647 (PMC13604818; doi:10.3390/biology15181647)
Supplement: Supplementary file 1 [file biology-15-01647-s001.zip › Data Related to “Spatial Variation in Stream-Water Bacterial Communities across Sites Differing in Wild Chinese Giant Salamander Detection Frequency”/Microbial Data/result/05.BetaDiversity/05.BetaDiversity_ReadMe.pdf]

# 扩增子结果文件补充说明（05）

2023 年 8 月 28 日

## 05.BetaDiversity【Beta 多样性分析结果】

### 1. beta_distance【Beta 距离分析结果】

1.1 sample/group1/ 【样本及组Beta 距离分析结果】

1.1.1 *.{sample，group}.txt【(binary_jaccard,bray_curtis,(un)weighted_unifrac) 距离值】

1.1.2 *.{sample，group}.pc.txt【(binary_jaccard,bray_curtis,(un)weighted_unifrac) PCoA分析主成分信息】

### 2. beta_upgam【beta upgam 分析结果】

2.1 sample/group1/ 【样本及组Beta UPGMA 分析结果】

2.1.1 (un)weighted_unifrac【基于(非)加权unifrac 距离 Beta UPGMA 分析结果】

2.1.2 (un)weighted_unifrac.{sample,group}_upgma.tre【基于(非)加权unifrac 距离的UPGMA 聚类树文件，MEGA 软件可以打开】

2.1.3 (un)weighted_unifrac.{sample,group}.{pdf,png} 【基于(非)加权unifrac 距离的UPGMA 聚类树图】

2.1.4 upgam.(un)weighted_unifrac.{sample,group}.tree.{svg,png} 【基于(非)加权unifrac 距离的UPGMA 聚类树合并图】

### 3. beta_pca【beta pca 分析结果】

3.1 sample/group1/ 【样本及组Beta PCA分析结果】

3.1.1 PCA.{xls,pdf,png}【PCA作图数据及未标样品名称的PCA图】

| 列数 | 列标题 | 含义 |
| --- | --- | --- |
| 1 | 样本名称 | 各样本的名称 |
| 2 | Axis1 | 1 维坐标 |
| 3 | Axis2 | 2 维坐标 |
| 4 | Axis3 | 3 维坐标 |
| 5 | Axis4 | 4 维坐标 |
| 6 | Axis5 | 5 维坐标 |

注：组内样本个数大于 3 时，才会生成置信椭圆

3.1.2 PCA_lable.{pdf,png}【标有样品名称的PCA图】

3.1.3 PCA_circle.{pdf,png}【未标样本名带有置信椭圆的PCA图】

3.1.4 PCA_circleLable.{pdf,png}【标有样本名带有置信椭圆的PCA图】

## 4. beta_pcoa【beta pcoa分析结果】

4.1 sample/group1/【样本及组Beta PCoA分析结果】

4.1.1 PCoA_display.{pdf,png}【基于(非)加权unifrac距离的PCoA合并展示图】

4.1.2 *【基于（(un)weighted_unifrac,binary_jaccard,bray_curtis）PCoA分析结果】

4.1.2.1 PCoA.{xls,pdf,png}【各样品在前两个主成分轴上的位置坐标及未标样品名称的PCoA图】

| 列数 | 列标题 | 含义 |
| --- | --- | --- |
| 1 | 样本名称 | 各样本的名称 |
| 2 | PCA1 | 1 维坐标 |
| 3 | PCA2 | 2 维坐标 |

注：组内样本个数大于 3 时，才会生成置信椭圆

4.1.2.2 PCoA_label.{pdf,png}【标有样品名称的PCoA图】

4.1.2.3 PCoA_circle.{pdf,png}【未标样本名带有置信椭圆的PCoA图】

4.1.2.4 PCoA_circleLabel.{pdf,png}【标有样本名带有置信椭圆的PCoA图】

4.1.2.5 emperor_pcoa_plots【三维PCoA网页版展示】

## 5. beta_nmds【beta nmds分析结果】

5.1 sample/group1/【样本及组Beta NMDS分析结果】

5.1.1 NMDS_display.{pdf,png}【基于(非)加权unifrac距离的NMDS合并展示图】

5.1.2 *【基于（(un)weighted_unifrac,binary_jaccard,bray_curtis）NMDS分析结果】

5.1.2.1 *.NMDS.{xls,pdf,png}【各样品在前两个主成分轴上的位置坐标及未标样品名称的NMDS图】

| 列数 | 列标题 | 含义 |
|---|---|---|
| 1 | 样本名称 | 各样本的名称 |
| 2 | NMDS1 | 1维坐标 |
| 3 | NMDS2 | 2维坐标 |

注：组内样本个数大于3时，才会生成置信椭圆

5.1.2.2 *.NMDS_lable.{pdf,png}【标有样品名称的NMDS图】

5.1.2.3 *.NMDS_circle.{pdf,png}【未标样本名带有置信椭圆的NMDS图】

5.1.2.4 *.NMDS_circleLable.{pdf,png}【标有样本名带有置信椭圆的NMDS图】

## 6. beta_dca【beta dca分析结果】

6.1 sample/group1/【样本及组beta dca分析结果】

### 6.2 DCA.{xls,pdf,png}【DCA 作图数据及DCA 图】

| 列数 | 列标题 | 含义 |
|---|---|---|
| 1 | 样本名称 | 各样本的名称 |
| 2 | Axis1 | 1 维坐标 |
| 3 | Axis2 | 2 维坐标 |

## 7. beta_unifracHeatmap【Beta unifrac 距离热图】

### 7.1 sample/group1/【样本及组Beta unifrac 距离热图】

#### 7.1.1 beta_diversity.heatmap.{svg,png}【包含(un)Weighted 两种距离的热图】

#### 7.1.2 beta_diversity.heatmap.(un)Weighted.{svg,png}【(非)加权unifrac 距离热图】

### 7.2 DCA.{xls,pdf,png}【DCA 作图数据及DCA 图】

## 8. beta_div【组间Beta 多样性比较箱型图】

### 8.1 group1【组间Beta 多样性比较箱型图】

#### 8.1.1 (un)weighted_unifrac【基于(非)加权unifrac 距离的组间Beta 多样性比较箱型图】

##### 8.1.1.1 (un)weighted_unifrac.{pdf,png}【基于(非)加权unifrac 距离的Beta 多样性箱型图】

##### 8.1.1.2 (un)weighted_unifrac_kruskalWallis_all.txt【多组间非参数kruskalWallis 检验（组内样本>2，组>2）】

| 列数 | 列标题 | 含义 |
|---|---|---|
| 1 | Difference | 均值差 |
| 2 | pvalue | P 值 |
| 3 | Signif. | 是否显著。若p 值<0.05,标*,p 值<0.01,标**, p 值<0.001,标*** |
| 4 | LCL | 置信区间下限 |
| 5 | UCL | 置信区间上限 |

8.1.1.3  8.1.1.3 (un)weighted_unifrac_kruskalWallis.{pdf,png}【多组间非参数kruskalWallis 检验可视化展示（组内样本>2,组>2）】

| 列数 | 列标题 | 含义 |
|---|---|---|
| 1 | diff | 组间差值 |
| 2 | lwr | 置信区间上限 |
| 3 | upr | 置信区间下限 |
| 4 | P adj | P 值 |

8.1.1.4 (un)weighted_unifrac_TukeyHSD_all.txt【多组间方差TukeyHSD 分析（组内样本>2，组>2）】

8.1.1.5 (un)weighted_unifrac_TukeyHSD.{pdf,png}【多组间方差TukeyHSD 分析可视化展示（组内样本>2，组>2）】

8.1.1.6 (un)weighted_unifrac_tTest_all.txt【组间T-test 检验（组内样本>2，组=2）】

| 列数 | 列标题 | 含义 |
|---|---|---|
| 1 | Group-pair | 比对组 |
| 2 | p_value | P值 |

8.1.1.7 (un)weighted_unifrac_tTest.{pdf,png}【组间T-test 检验可视化展示（组内样本>2，组=2）】

8.1.1.8 (un)weighted_unifrac_twoWilcox_all.txt【组间twowilcox 秩和检验（组内样本>2，组=2）】

| **列数** | **列标题** | **含义** |
|---|---|---|
| 1 | Group-pair | 比对组 |
| 2 | p_value | P值 |

8.1.1.9 (un)weighted_unifrac_twoWilcox.{pdf,png}【组间twowilcox 秩和检验可视化展示（组内样本>2，组=2）】
